# A year of contemplation: looking back and moving forward

**DOI:** 10.1186/1757-7241-17-31

**Published:** 2009-07-13

**Authors:** Kjetil Søreide, Hans Morten Lossius

**Affiliations:** 1 Department of Surgery, Stavanger University Hospital, Stavanger, Norway; 2 Department of Surgical Sciences, University of Bergen, Bergen, Norway; 3 Norwegian Air Ambulance, Drøbak, Norway

## Editorial

As we enter July 2009, we are pleased to have passed the first 365 days of being an open access, freely available online journal on the World Wide Web and indexed in Pubmed. Currently 49 papers have been published and several more are awaiting publication in the near future. We experience a steady and sound inflow of new manuscripts submitted to the Journal, and are in the position to select those deemed to be of most value, with a rejection rate above 50% at present. Indeed, based on the understanding that management of emergencies, injuries and critically ill patients is multidisciplinary team-work, the Scandinavian Journal of Trauma, Resuscitation and Emergency Medicine (SJTREM) has not only matured[1] and grown up[2], it has also found its place in the joint area of trauma, resuscitation and emergency medicine. Those who think that the year of success would lead us to rest on the laurels of the past should think again. We envision further growth and aim for higher standards and call for increased multidisciplinary input to the Journal in the years to come!

The Journal has over the past year received and published original and review papers from several Scandinavian research groups [3-11]. Also, contributions from several international researchers including the USA [12-15], several European countries (e.g. UK, Germany, Switzerland, Italy and Greece) [16-22], and even Asia [23] are influencing the Journal content. Currently, we have submissions in-house from virtually all corners of the world, with several contributors outside Scandinavia and Europe. We believe this demonstrates the increasing global impact of the Journal and hope to establish a truly international profile and outreach while not loosing the vision of being a forum for scientific exchange and improvements for Scandinavian countries in particular. Also, the Journal has supported and published abstracts from international congresses, with the supplements from the *London Trauma Conference *available on the website[24], and the abstracts for the *3rd Scandinavian Update on Trauma, Resuscitation and Emergency Medicine *and the *Research Symposium 2009 for the Danish Society of Emergency Medicine *will follow soon.

The success of the Journal is measured also by the number of accessed papers – currently, more than 10 of the published papers have been accessed over 1000 times, even though many of them have been available for only a few months on the Web, and 3 papers have been accessed more than 2000 times each[13,25,26]. The international consensus paper by Ringdal et al[26] has even been included in the "Faculty of 1000 medicine" with almost 5000 hits in less than one year.

We strive to ensure high visibility of the Journal, and have for 2008 published a yearbook, which has been distributed to several hundred participants at the *Scandinavian Update conference *as well as to a wide number of libraries and research institutions. The success of this publication will be repeated for 2009, thus ensuring high visibility both electronically as well as on paper.

Several of the current progresses and developments will hopefully ensure SJTREM a proper index in MEDLINE and indexing in the Journal Citation Report©, which will provide us with an official impact factor (IF) in the near future. We are confident that the time the IF is released it will reflect an already highly visible and dynamic forum for publication in trauma, resuscitation and emergency medicine. However, knowing the success of any journal is only dependant on three factors – the authors, the peer-reviewers and notably the readers – we want to forward our sincere thanks to those contributing their efforts, sharing their thoughts and devoting their time to ensure the scientific and educational value of the Journal. All peer-reviewers will be appropriately acknowledged in the paper version of the yearbook. For those of you interested in the yearbook, please contact editorial@sjtrem.com. We would also welcome the readers' feedback on the Journal – be it positive or negative.

## Authors' information

Kjetil Søreide and Hans Morten Lossius are Editors-in-Chief of Scandinavian Journal of Trauma, Resuscitation and Emergency Medicine
